# Rifabutin-Induced Thrombocytopenia in a Patient With Uncontrolled HIV: A Case Report

**DOI:** 10.7759/cureus.66339

**Published:** 2024-08-06

**Authors:** Amer Tamr, Dana Kabbani, Jarrett J Weinberger

**Affiliations:** 1 Internal Medicine, Henry Ford Health System, Detroit, USA; 2 Internal Medicine, Wayne State University School of Medicine/Detroit Medical Center, Detroit, USA; 3 Internal Medicine, Wayne State University/Detroit Medical Center, Detroit, USA

**Keywords:** immune-mediated thrombocytopenia, medication-induced thrombocytopenia, severe thrombocytopenia, disseminated mycobacterium avium complex disease, uncontrolled hiv

## Abstract

Uncontrolled HIV is associated with a wide range of hematologic abnormalities through direct suppressive effects, opportunistic infections, tumor marrow infiltration, or antiretroviral, antimicrobial, or antitumor therapy. We present a patient with a history of uncontrolled HIV presenting with acute severe thrombocytopenia shortly after starting treatment for disseminated Mycobacterium avium complex (MAC). While the thrombocytopenia was resistant to transfusion and intravenous immunoglobulin (IVIG), it mildly improved with dexamethasone after holding home medications. Etiologies for this patient's thrombocytopenia include uncontrolled HIV infection and medication-induced, likely secondary to rifabutin. We propose a possible combined effect of both factors. Clinicians should be aware of the increased risk of severe, acute medication-induced thrombocytopenia in patients with uncontrolled HIV, given their baseline susceptibility to hematologic abnormalities.

## Introduction

Uncontrolled HIV is associated with hematologic abnormalities ranging from isolated anemia, lymphopenia, granulocytopenia, or thrombocytopenia to pancytopenia. Thrombocytopenia occurs in approximately 10-50% of patients with HIV as one of the first clinical signs of infection [1]. A study of 1004 HIV-infected patients showed that thrombocytopenia was more common in white males and older subjects, as well as in patients with acquired immunodeficiency syndrome (AIDS), CD4 cell numbers <200/mm³, and advanced disease [2]. The pathophysiology likely involves the direct suppressive effects of HIV, opportunistic infections, tumor marrow infiltration, or antiretroviral, antimicrobial, or antitumor therapy [1]. Medication-induced thrombocytopenia in patients with HIV occurs through both immune-mediated and non-immune-mediated mechanisms [3]. The former involves the formation of drug-dependent antibodies that target platelet-specific glycoproteins. Platelet-antibody complexes are then destroyed through complement activation or direct lysis. The latter involves medications causing direct toxicity to platelets and precursor cells, impairing platelet production by affecting the bone marrow, or directly inhibiting megakaryocyte function [4]. Antiretroviral medications commonly implicated in thrombocytopenia include protease inhibitors Lopinavir and Ritonavir [5]. Determining the primary cause of thrombocytopenia in these patients is critical for guiding treatment. This case follows a patient with a history of poorly controlled HIV presenting with acute severe thrombocytopenia.

## Case presentation

A 42-year-old man with a past medical history of uncontrolled HIV with a CD4 count of 15/mm³ and viral load (VL) of 2052 copies/mL presented to the hospital for severe thrombocytopenia with associated epistaxis and petechiae. He reported nose bleeding that started for the first time that day. He had been discharged five days prior with azithromycin, trimethoprim-sulfamethoxazole, ethambutol, and rifabutin for disseminated Mycobacterium avium complex (MAC), and emtricitabine/tenofovir and dolutegravir for known HIV. On physical exam, the patient’s nostrils were packed. Laboratory studies revealed a platelet count of 1000 platelets/μL.

Given that he recently began MAC treatment, drug-induced thrombocytopenia was suspected, and all MAC treatment medications were held. HIV medications were continued because the patient had previously been on the same antiretroviral therapy (ART) without complications in the past. Stopping individual MAC treatment medications in a step-wise fashion to possibly reveal the culprit was considered. Another consideration was the risk of complications associated with severe thrombocytopenia, particularly in the setting of possible immune thrombocytopenic purpura (ITP), including intracranial hemorrhage (ICH). Given the severity of the thrombocytopenia, unclear etiology, and concern for ICH, all medications were stopped at once.

Bone marrow biopsy showed megakaryocytes consistent with immune-mediated thrombocytopenia either secondary to ITP or drug-induced (DITP). Drug-dependent platelet antibody (antiplatelet antibody) testing was ordered but did not return during the inpatient course. Parvovirus antibodies returned negative. Thrombocytopenia was resistant to four platelet transfusions and two doses of intravenous immunoglobulin (IVIG).

It was difficult to make the distinction between ITP and DITP, but concern for ITP grew given the lack of improvement in platelet count after six days despite stopping recently initiated medications. As such, the patient was started on dexamethasone 40 mg daily for four days for treatment of ITP. Platelet count mildly improved with dexamethasone and was 70,000 platelets/μL by discharge, 10 days after the first dose.

**Figure 1:**
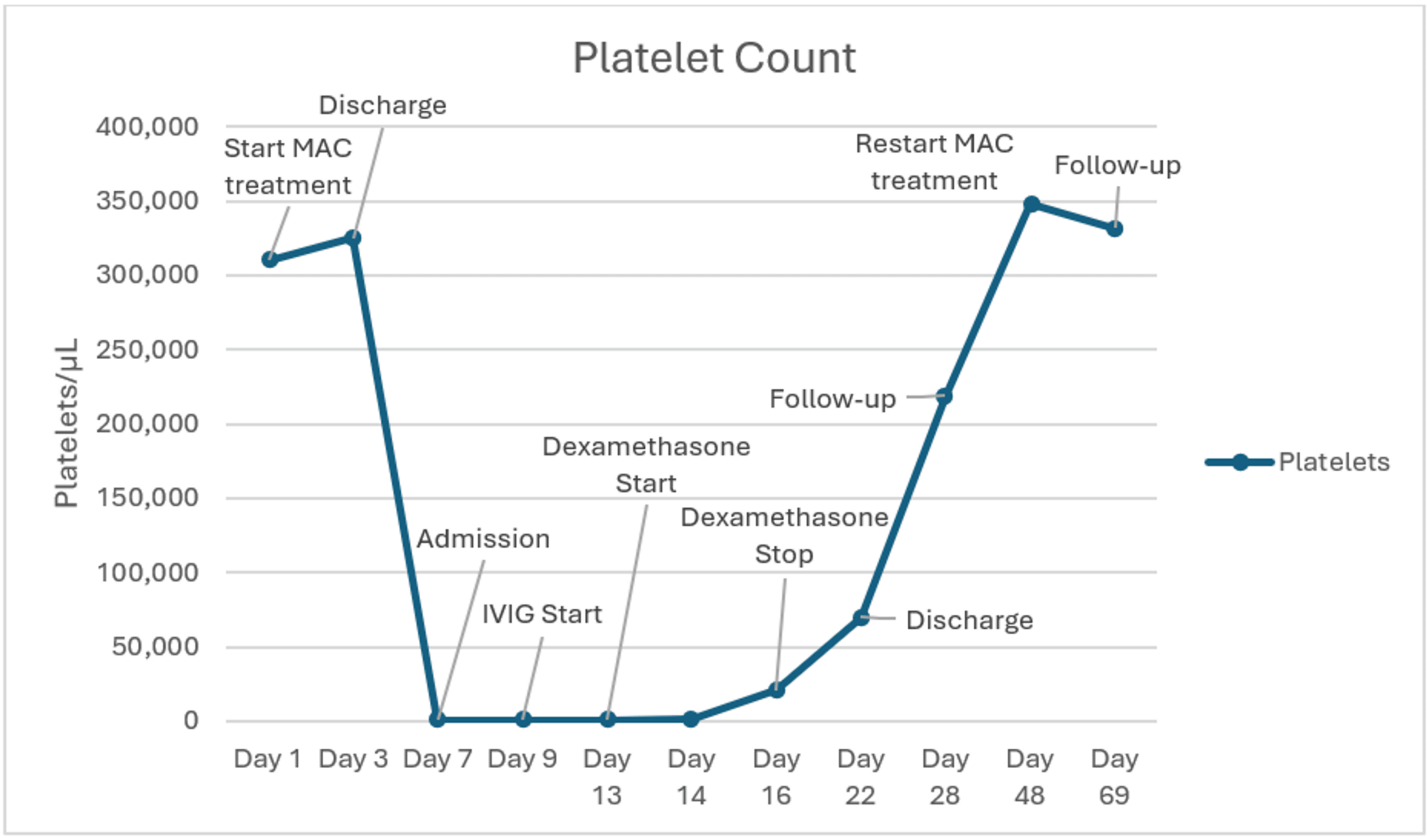
Platelet count trend from initiation of MAC treatment until outpatient follow-up MAC = Mycobacterium avium complex

At follow-up one week after discharge, platelet count was 219,000 platelets/μL and VL 162 copies/mL, indicating viral suppression. At that time, testing for antiplatelet antibodies associated with ethambutol and trimethoprim-sulfamethoxazole, medications known to be implicated in DITP, had returned and showed negative results. Thus, all MAC treatment was restarted at once (Figure 1). Starting medications in a step-wise fashion and monitoring for refractory thrombocytopenia was considered. However, given the patient's recent hospitalization for MAC infection and that treatment had been held for three weeks due to thrombocytopenia, prompt re-initiation of full treatment was prioritized. The patient continued to have regular follow-ups every six months without refractory thrombocytopenia. He also remained virally suppressed. Platelet count has remained stable for five years.

## Discussion

Our literature review is significant for patients with uncontrolled HIV experiencing thrombocytopenia [1]. Further review reveals patients experiencing thrombocytopenia during long-term treatment of MAC disease with antimycobacterial medications. A retrospective study found that 28.6% of patients being treated for MAC lung disease experienced thrombocytopenia with a median time of onset of 61.5 days from treatment initiation [6]. The literature also demonstrates a case of thrombocytopenia occurring within five days of initiating treatment with azithromycin [7]. Ethambutol has also specifically been implicated in cases of thrombocytopenia while being administered with other antituberculosis medications [8]. Furthermore, rifabutin-induced thrombocytopenia has widely been reported in the literature and is thought to have a dose-dependent effect [9].

DITP occurs via suppression of platelet production or immunologic platelet destruction, the latter being more common. Platelets form medication-antibody complexes that are then damaged by complement activation [10]. Considering antiplatelet antibody testing associated with our patient's medications was negative, the most likely underlying mechanism of his DITP would be suppression of platelet production. However, the acute drop in platelet count within days of initiating MAC treatment and lack of improvement with transfusions makes it a less likely explanation and favors an immune-mediated destructive process. Improvement after a short course of dexamethasone favors an immune-mediated process as well [11].

Clinical criteria used to aid in the diagnosis of DITP include exposure to new drugs 5-10 days prior to thrombocytopenia onset, recovery after discontinuation of the culprit drug, continuation or reintroduction of other drugs after discontinuing culprit drug with sustained platelet count, exclusion of other causes of thrombocytopenia, and re-exposure to the drug causing recurrent thrombocytopenia [12]. Our patient met at least two of the DITP diagnostic criteria, but it is thought that his underlying uncontrolled HIV complicated the picture as it was a likely contributor to the thrombocytopenia and slowed the rate of improvement after discontinuing the culprit drug. Gaining control over the HIV infection was suspected to be necessary to see improvement and was thought to have reduced the patient's risk for DITP, allowing the reintroduction of MAC treatment without recurrent thrombocytopenia. This argument is rooted in the fact that the only variable that changed between the patient's initial presentation with thrombocytopenia and his follow-up appointments was his HIV control, as measured by viral load. All medications remained unchanged.

Stepwise discontinuation of MAC treatment medications may have been helpful in determining the culprit medication in our case. However, given the severely low platelet count of unclear duration at presentation and concern for ICH in the setting of possible ITP, all medications were stopped simultaneously without de-challenge testing [13]. Reinitiating medications in a stepwise fashion (rechallenge test) at follow-up could have served as an alternative means to determine the responsible medication, but was not performed by the responsible provider.

It is suspected that rifabutin was responsible for inducing thrombocytopenia in our patient given negative drug-dependent antibody testing for ethambutol and trimethoprim/sulfamethoxazole and lower likelihood of azithromycin involvement as the patient had been treated with it in the past without complication. Given the severity of our patient's thrombocytopenia on presentation, we hypothesize a combined effect of uncontrolled HIV infection and rifabutin-induced thrombocytopenia. This conclusion is supported by the absence of thrombocytopenia after restarting the same MAC treatment regimen upon achieving viral suppression. Conclusions are limited by a lack of de-challenging or rechallenging medications to assist with clinically determining the causative agent. They are also limited by the inability to test for all medication-associated antibodies as laboratories do not typically test for less common antiplatelet antibodies.

## Conclusions

Despite widespread access to HIV preventative therapies in the modern era, it remains a prevalent virus and often goes undertreated. Uncontrolled HIV is associated with hematologic abnormalities, including thrombocytopenia. Patients with uncontrolled HIV are at significant risk of contracting other infections, including MAC, which can either directly cause or may require treatment that can cause or exacerbate hematologic abnormalities. Clinicians should be aware of the increased risk of severe, acute DITP in patients with uncontrolled HIV, given their baseline susceptibility to hematologic abnormalities. Furthermore, although it is not required for diagnosis of DITP, clinicians should consider advanced testing for drug-dependent antibodies in select, high-risk populations, such as HIV patients, to ensure safety when selecting medication regimens.
